# Evaluation of at-home serum anti-Müllerian hormone testing: a head-to-head comparison study

**DOI:** 10.1186/s12958-022-01004-2

**Published:** 2022-09-01

**Authors:** Erin Silliman, Esther H. Chung, Elizabeth Fitzpatrick, Julie A. Jolin, Michelle Brown, James Hotaling, Aaron K. Styer, Anatte E. Karmon

**Affiliations:** 1 TLC Infertility & Donor Services, 1920 Hillhurst Ave, Los Angeles, CA 90027 USA; 2 Stanford Fertility and Reproductive Health Services, 1195 W Fremont Ave, Sunnyvale, CA 94087 USA; 3 Massachusetts General Hospital, 55 Fruit St, Boston, MA 02114 USA; 4 Northwestern Women’s Hospital, 250 E Superior St, Chicago, IL 60611 USA; 5 University of Utah 201 Presidents’ Cir, Salt Lake City, UT 84112 USA; 6 CCRM Fertility Boston, 300 Boylston Street, Chestnut Hill, MA 02459 USA; 7 Fertility Institute of Hawaii, 1401 S Beretania St Suite 250, Honolulu, HI 96814 USA

**Keywords:** Anti-Müllerian hormone, AMH, Home fertility testing, Blood collection, Home device

## Abstract

**Background:**

For optimal fertility testing, serum anti-Müllerian hormone levels are used in combination with other testing to provide reliable ovarian reserve evaluations. The use of the ADx 100 card is widely commercially available for at-home reproductive hormone testing, but data demonstrating that its results are reproducible outside of a clinical setting are limited, as well as comparisons of its performance with other newer blood collection techniques. This study aimed to evaluate the concordance of serum AMH levels found via standard venipuncture and self-administered blood collection using the TAP II device (TAP) and ADx card in women of reproductive age.

**Methods:**

This was a prospective, head-to-head-to-head within-person crossover comparison trial that included 41 women of reproductive age (20–39 years). It was hypothesized that the TAP device would be superior to the ADx card both in terms of agreement with venipuncture reference standard and patient experience. Each subject had their blood drawn using the three modalities (TAP, ADx, and venipuncture). We evaluated the concordance of AMH assays from samples obtained via the TAP device and ADx card with the gold standard being venipuncture. Two-sided 95% CIs were generated for each method to compare relative performance across all three modes. Patient preference for the TAP device versus the ADx card was based on self-reported pain and Net Promoter Score (NPS).

**Results:**

The TAP device was superior to the ADx card on all outcome measures. TAP R-squared with venipuncture was 0.99 (95% CI 0.99, > 0.99), significantly higher than the ADx card, which had an R-squared of 0.87 (95% CI 0.80, 0.94) under most favorable treatment. TAP sensitivity and specificity were both 100% (no clinical disagreement with venipuncture), versus 100 and 88%, respectively, for the ADx card. Average pain reported by users of the TAP device was significantly lower than the ADx card (0.75 versus 2.73, *p* < 0.01) and the NPS was significantly higher than the ADx card (+ 72 versus − 48, *p* < 0.01).

**Conclusions:**

The TAP was non-inferior to venipuncture and superior to the ADx card with respect to correlation and false positives. Moreover, the TAP was superior to both alternatives on patient experience.

**Trial registration:**

NCT04784325 (Mar 5, 2021).

**Supplementary Information:**

The online version contains supplementary material available at 10.1186/s12958-022-01004-2.

## Background

Due to the rapid emergence of telehealth and digital health, an increasing number of patients and their providers are taking advantage of at-home diagnostic testing options. Women for years have been able to test different aspects of their fertility using over-the-counter tests measuring markers of ovulation, pregnancy, and menopause [1]. There also exists a multitude of at-home dried blood spot (DBS) tests that analyze saliva, urine, serum, and other samples to test for sexually transmitted infections and hormone levels [2]. The use of the DBS ADx card is popular for at-home fertility and hormone testing due to its reported accuracy [2, 3]; however, data demonstrating that the results are reproducible in real world conditions are limited by insufficient validation data and small sample sizes [4, 5].

The combination of delayed family building and age-related fertility decline highlights the current need for reliable and accessible fertility testing options [6]. Particularly given the intimate nature of fertility care and the high prevalence of infertility, increasing the awareness of and access to basic fertility testing seems prudent. With access to fertility testing at home, women have reported feeling more empowered, excited, and prepared, and less anxious and confused, regarding their fertility status [7].

During initial fertility testing, serum anti-Müllerian hormone (AMH) levels are used in combination with other testing to provide ovarian reserve evaluation [8]. The ability to do this accurately by using the most accurate collection device [9], at home, has the potential to improve access to high-quality fertility care and identify patients who would most benefit from further infertility specialist (REI) consultation. To this end, our study aimed to assess the concordance of AMH levels found in blood via standard venipuncture and self-administered blood collection using two possible methods for at-home collection: the TAP II device (TAP) and ADx card in women of reproductive age. It was hypothesized that the TAP device could be superior to the ADx card for the purpose of AMH collection, both in terms of agreement with a venipuncture reference standard and patient experience. The study design itself, however, was agnostic as to the direction of differences (if any) that might exist between the devices on these dimensions.

## Materials and methods

### Protocol and overview

The AMH^2^ study [Anti-Müllerian Hormone – At My Home (AMH^2^)] was a head-to-head-to-head within-person crossover trial conducted in Boston, Massachusetts. This study design was selected because each subject serves as their own control, effectively reducing inter-subject variation and improving statistical power. The different serum tests were completed sequentially during the same session, ensuring the samples were directly comparable with no further need for randomization. Institutional research ethics board (IRB) approval was obtained from Ethical & Independent Review Services IRB, a third-party IRB accredited by the Association for the Accreditation of Human Research Protection Programs [10]. The device examined in this trial was the TAP II by YourBio Health (formerly Seventh Sense Biosystems). The trial was registered on ClinicalTrials.gov as NCT04784325 [11]. No changes were made to the methods after commencement of the trial.

### Collection devices

**Fig. 1:**
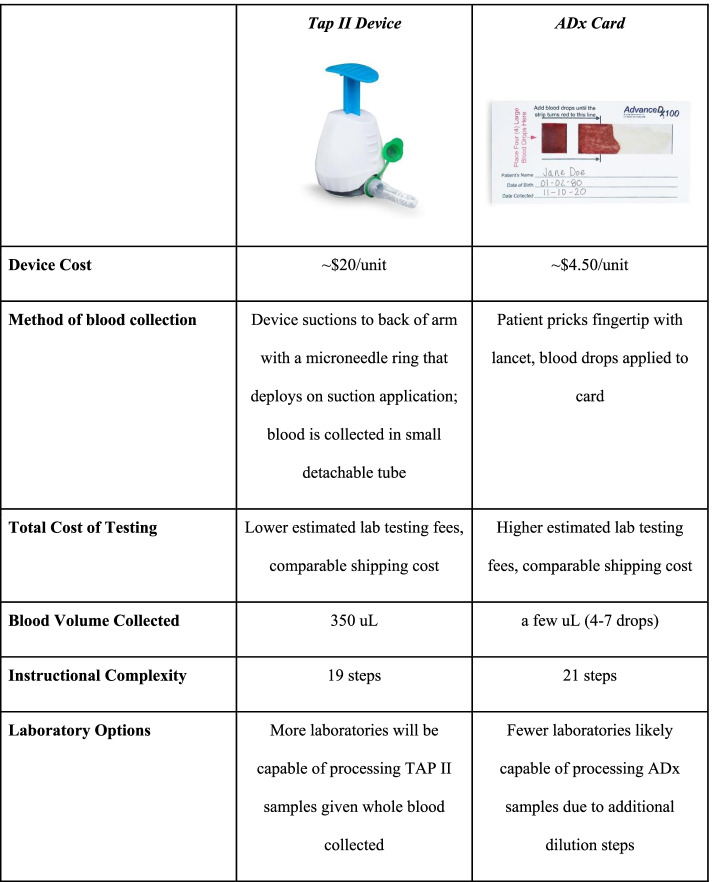
Device Comparison

Figure 1 outlines the differences between these two devices. The TAP device is a small unit that attaches via suction to the back of the patient’s arm and uses a microneedle array to pierce capillaries close to the surface of the skin, collecting the specimen in a vial that then detaches from the device. In comparison, the ADx card collects a few drops of blood that a patient obtains from a lanced fingertip, and blood is then diluted during lab processing for analysis.

The most notable clinical difference between devices are the collection of whole blood (TAP) versus dried blood (ADx), the use of microneedles (TAP) versus finger-prick (ADx).

### Recruitment and study procedure

Recruitment utilized social media platforms, email recruitment messages, announcements within women’s professional networks, referrals from physicians on Turtle Health’s Medical Advisory Board, and outreach to subjects in Turtle Health’s SELF-HELP (Sonograms Enable Looking Forward – Home Examinations Led by Providers) ultrasound validation study [12]*.* Subjects were all women between the ages of 20 and 39 (inclusive), able to freely give consent electronically, spoke native or fluent English, had a high school degree or equivalent, and lived within driving distance from Boston. Sponsor employees, women who had recently give birth or had fewer than 3 postpartum menstrual cycles, were currently pregnant or possibly pregnant, and with known bleeding disorders or coagulopathies were excluded.

In total, 69 women were screened, and 41 participated. Of the 28 patients who were screened but not enrolled, 7 were unable to enroll due to inadequate space in their age cohort, 8 were in the screening process when the study completed, 7 never responded after receiving a consent form electronically, and 1 was not eligible because she did not live nearby. An additional 8 women were found to not meet study inclusion criteria. 5 women withdrew after signing the consent form but prior to the start of the trial, with reasons including contracting COVID-19, scheduling difficulties, and failure to respond to follow-up. An additional 7 women who expressed interest in the study never completed the screening questionnaire provided.

This number of participating subjects (*N* = 41) was over two times the sample size per the Clinical Laboratory Improvement Amendments (CLIA) lab requirements, and provided over 80% power to detect a one-half standard deviation difference in device performance. Inclusion criteria included healthy women aged 20–39 who were able to consent electronically given COVID, which for the purposes of this study was defined as those who speak native or fluent English, have a high school degree or equivalent, and were within driving distance of Boston. Exclusion criteria included Turtle Health employees, women who did not speak English natively or fluently, and postpartum women who had fewer than 3 postpartum menstrual cycles.

For each subject, the study consisted of a single visit to a medical office where blood was drawn using three modalities sequentially in the same ~ 30-minute session: two self-administered TAPs, one self-administered ADx card, and one venipuncture vial drawn by a professional phlebotomist. For both TAP and ADx card samples, participants were instructed to follow the written patient labeling and received no additional verbal direction by the trial coordinator outside of trial-specific instructions. The draw order was consistent for each participant. Samples were de-identified of patient data and labeled with assigned identification numbers. One TAP for each woman was 2-day shipped to the processing lab via the United Parcel Service (UPS®) in Turtle Health’s commercial packaging to simulate the shipping process that would be required of at-home consumers should this product reach the market. All other samples (remaining TAP, ADx card, and venipuncture vial) were hand-delivered to the lab within 6 hours of the blood draw. The venipuncture sample served as the reference standard for AMH for each subject and was processed by the lab upon receipt. Both TAPs and ADx samples were processed by the lab at t = 72 hours.

Samples were processed at BioAgilytix, an independent, Boston-based laboratory, using the Roche Elecsys® AMH assay [13]. One shipped TAP sample, two non-shipped TAP samples and one ADx card were designated “quantity not sufficient” (QNS) for processing by the lab. These 4 QNS results out of 164 assays were not included in the following analyses. Other non-QNS data from subjects with a QNS results remain in the results other than for pairwise comparisons, which consist of only pairs where both results were obtained.

### Favorable treatment for the ADx comparator device

The ADx 100 card was chosen as a comparator to the TAP device given its widespread use in home AMH analysis kits. As the current standard of care, ADx 100 card was subject to the most favorable treatment possible throughout study setup, testing, and lab processing to enhance its clinical relevancy.

ADx cards were stored and processed in accordance with manufacturer instructions. Part of those instructions include a lab-dependent correction factor on test results, which typically read much lower than whole-blood samples. To ensure most favorable treatment, the most generous assumptions plausible under manufacturer guidance were applied: first by precisely normalizing ADx results to venipuncture to remove any directional bias (a ~ 16.2x dilution factor, calculated with the trial data itself), and then by simulating an ideal total protein correction. Typically, the final result of an ADx AMH blood test is adjusted by a factor based on total protein. Manufacturer guidance suggested that this factor was no higher than a 15% adjustment for 99.9% of samples, but can vary by lab. To obtain the best theoretically possible result, every normalized ADx sample was adjusted to be 15% closer to the precise venipuncture result (or adjusted to be equal to the venipuncture result, if the difference between the two was < 15% of the normalized ADx result). For example, if venipuncture obtained a 1.20 ng/mL reading and the ADx card obtained a 1.00 ng/mL reading after dilution adjustment, the ADx result was adjusted to 1.15 ng/mL for the purpose of the analysis.

### Statistical methods

Analysis was performed using the 2019 version of Microsoft Excel [14]. Results from the ADx card were adjusted to ensure most favorable treatment under manufacturer guidance as explained above. Results obtained from the shipped TAP device were all reduced by a constant ~ 5.6%, as AMH increases slightly in stored blood samples over time and requires an experimental correction to remove bias from the assay [15]. Given that minor variations of AMH levels due to shipping times have little impact on clinical categorization and as the TAP devices were consistently shipped via a 2-day guaranteed courier service, a constant correction factor was deemed sufficient for the purposes of this comparison study [16]. Similar to the ADx results, this factor was also calculated in-sample based on the observed average difference between venipuncture and the shipped TAP device. This estimate was consistent across samples, with a standard deviation of only 0.035.

The primary endpoint of the study was the correlation of AMH concentration obtained via the gold standard of venipuncture with the TAP device and the ADx card, respectively, demonstrating each device’s ability to replicate per-person findings that would have been obtained in-clinic. Prior to the main study, precision validation showed that both intra and inter-assay precision were within 1.69% across 65 replicates. Given this consistency, venipuncture on this assay was treated as the reference standard in the remainder of the trial. No changes were made to the trial outcomes after the trial commenced.

To assess the likely patient impact of the use of each device, categorical agreement of results was examined. Using previous literature and published nomograms, practicing physicians pre-defined age and hormonal birth control adjusted thresholds for results, so that each AMH result was categorized as either “Very low” (<5th percentile), “Low” (5th–10th percentile), “Normal” (25th–75th percentile), or “High” (>75th percentile) [17–19]. The rate of agreement between categories across collection methods was then calculated. Furthermore, results below the 10th percentile for the subject’s age and birth control status were deemed to be clinically significant, as they would typically lead to a referral to a reproductive endocrinologist (REI).

Age-based percentiles were determined based on previous studies [17, 18]. However, these large-scale studies of population AMH levels did use different assays than that used in this trial. As such, they may not reflect the true percentile of a subject, but do provide an external source for results that are potentially clinically relevant in a broad population. Additionally, as the populations represented in both the Shebl et al. (2011) and Tehrani et al. (2014) studies were not on hormonal birth control, and because hormonal birth control is known to reduce AMH levels in the body, percentiles for patients on hormonal birth control were adjusted (lowered) using a factor derived from the Birch Peterson et al. (2015) study [19]. Positive predictive value and negative predictive value for each device (given this threshold) were calculated, as well as the false positive and false negative rates. False positive results occur when a patient is wrongly identified as having an AMH result lesser than the tenth percentile, triggering a referral to a REI when not otherwise necessary. False negative results occur when a patient is wrongly identified as having an AMH result greater than the tenth percentile, in which case they may miss out on a timely assessment by a REI.

Patient preference was also examined. The direct discomfort from the blood draw was assessed using the NRS-11 Pain Scale, a validated, single dimension, 0–10 scale, with clarifying benchmarks to help respondents identify their relative levels of pain [20, 21]. For example, a 0 indicates “No pain. Feeling perfectly normal.” and a 3 indicates “Very noticeable pain, like an accidental cut, a blow to the nose causing a bloody nose, or a doctor giving you an injection.” Scores were aggregated and averaged across each modality.

Overall patient experience was assessed using Net Promoter Score (NPS), an established and validated measure of preference in a variety of domains, both commercial and clinical. Net Promoter Score measures customer satisfaction with a product or experience [22]. When completing the measure, respondents are asked: “How likely are you to recommend this product to a friend or colleague?” Scores of 9–10 are promoters, 6 and below are detractors, and 7–8 are neutral. Percent detractors are then subtracted from percent promoters; the score range is from − 100 (all detractors) to + 100 (all promoters). The Net Promoter Score indicates a patient or consumer’s satisfaction with an experience compared to plausible alternatives.

## Results

### Participant characteristics

Forty-one participants were recruited and enrolled between April and June of 2021, providing four samples each and completing a follow-up survey. Forty of the forty-one participants completed their follow-up survey. The participants ranged in age between 21 and 39 years, averaging 27.8 years. The mean body mass index (BMI) for participants was 23.4 kg/m2, and 28% of the subjects’ BMIs were greater than 25 kg/m2 (overweight). The study comprised of 65% white subjects and 35% non-white/minority subjects including black, Asian, and Hispanic, within the catchment area’s reported range of ethnic diversity (26–40%) [22]. Contraceptive methods used by participants included hormonal (e.g. Mirena, Kyleena) and nonhormonal (e.g. Paragard) intrauterine devices (IUDs) (35%), oral contraceptives (20%), NuvaRing (8%), Implant (3%), or no contraceptive (35%). The majority of study participants self-reported their menstrual intervals to fall between 22 and 35 days, with 8% reporting greater than 35 days, 5% less than 22 days, and 23% unsure of their average menstrual intervals (see Table 1).

**Table 1:** Participant characteristics. Number of participants (*N* = 40) is less than the total enrolled in study (*N* = 41) as one participant did not complete their post-exam survey

Characteristic	Trial population, n (%) (***N*** = 40)
Age, mean (range)	28.7 (21, 39)
Race/Ethnicity	
White	26 (65%)
Non-white, minority	14 (35%)
BMI, kg/m^2^, mean (range)	23.4 (17.3, 29.6)
BMI > 25 (overweight)	11 (28%)
Contraception method	
Oral contraceptives	8 (20%)
IUD	14 (35%)
NuvaRing	3 (8%)
Implant	1 (3%)
None	14 (35%)
Menstrual interval	
22–35 days	26 (65%)
> 35 days	3 (8%)
< 22 days	2 (5%)
“Unsure”	9 (23%)

**Fig. 2:**
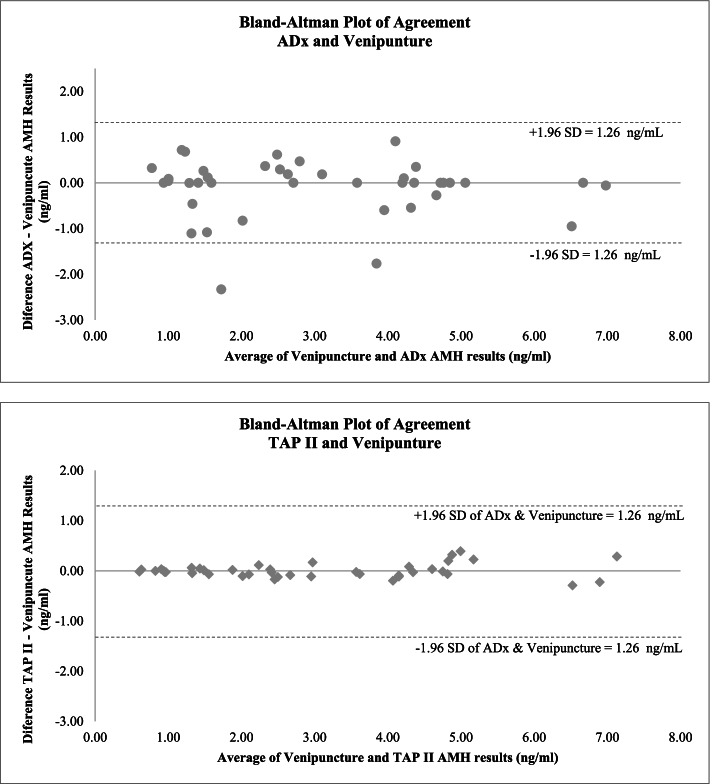
Bland-Altman plots of difference in AMH values by average values measured by each challenge device versus venipuncture (ng/dL)

The AMH range produced by venipuncture among the participants was 0.61–7.01 ng/dL, with a median of 2.56 ng/dL and a mean of 3.02 ng/dL. Bland-Altman plots of agreement of the test devices with venipuncture across this range are provided in Fig. 2.

### Correlation

**Fig. 3:**
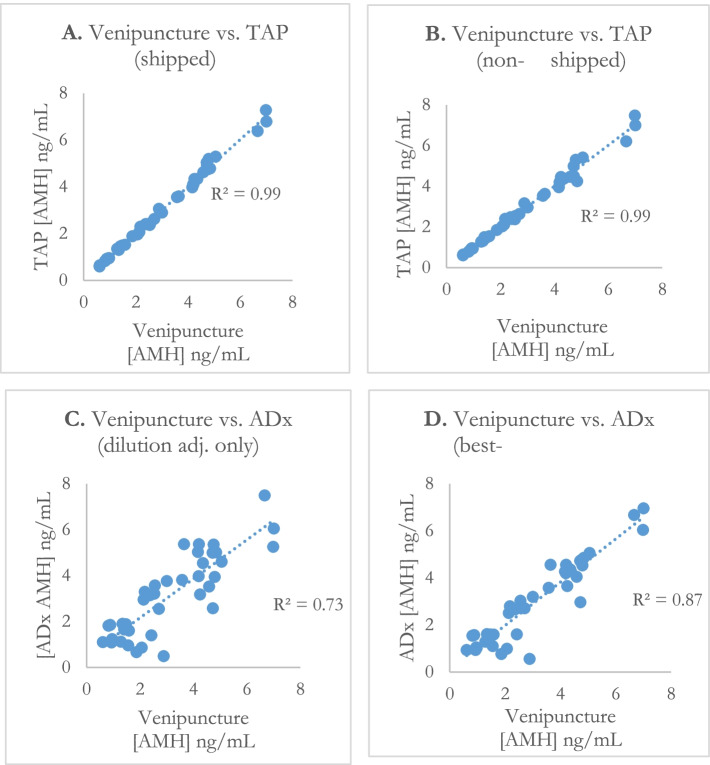
Measured AMH concentration vs. venipuncture and R-squared. AMH results via venipuncture sample versus shipped TAP II Device sample (**A**). AMH results via venipuncture sample versus non-shipped TAP II Device sample (**B**). AMH results via venipuncture sample versus ADx card sample adjusted with ~ 16.2x dilution factor (**C**). AMH results via venipuncture sample versus ADx card incorporating best-case adjustment factors (**D**)

The primary endpoint of the study is the correlation of AMH levels between the shipped TAP samples and venipuncture samples (Table 2, Fig. 3). The obtained R-squared was 0.99 (95% CI 0.99, > 0.99), demonstrating extremely strong concordance between Turtle Health’s shipped blood product and an in-clinic draw. The correlation of AMH levels between the 40 stored TAP samples vs. venipuncture was similarly high, with an R-squared of 0.99 (95% CI 0.98, 0.99). Root-mean-square deviation (RMSD) from venipuncture was low both with (0.14) and without (0.26) the empirical 5.6% correction factor. The correlation between the TAP II shipped vs. non-shipped was 0.994, indicating that the intra-patient reproducibility was very high.

**Table 2:** Performance of at-home blood collection devices for anti-Müllerian hormone

	TAP (shipped)	TAP (non-shipped)	ADx (w/o correction)	ADx (best-case correction)	Venipuncture
Correlation to venipuncture	0.99 [95% CI 0.99, > 0.99]	0.99 [95% CI 0.98, 0.99]	0.73 [95% CI 0.59 to 0.87]	0.87 [95% CI 0.80 to 0.94]	–
False-positive	0	0	4	4	–
False-negative	0	0	3	0	–
Sensitivity	100%	100%	57%	100%	–
Specificity	100%	100%	88%	88%	–
NRS-11 pain scale	0.75 [95% CI 0.53 to 0.97]		2.73 [95% CI 2.42 to 3.03]		1.85 [95% CI 1.49 to 2.21]
Net Promoter Score (NPS)	+ 72 [95% CI + 53 to + 90]		−48 [95% CI − 72 to − 23]		+ 20 [95% CI − 6 to + 47]

The correlation of AMH levels from the ADx card vs. venipuncture was significantly lower, both unadjusted (R-squared 0.73, 95% CI 0.59, 0.87) and after incorporating all best-case correction factors (R-squared 0.87, 95% CI 0.80, 0.94), with a best-case RMSD (root-mean-square deviation) of 0.64. The 95% CI for this best-case correction fell entirely below the lower bound for the TAP device’s 95% CI. An analysis of the same data using a mixed model demonstrated similar results: while both the ADx card and TAP II device were significantly correlated with venipuncture, the standard error of the estimate with the adjusted ADx card was 28% higher than that obtained with the TAP II device.

Overall, the shipped TAP device provided results that matched the percentiles ranges calculated based on venipuncture results in 97.5% of complete samples while the non-shipped TAP device showed 100% agreement. The same was observed for only 85% of complete samples for the ADx card. Both the shipped TAP device and ADx card had a single sample that had insufficient volume for analysis and were not included in these calculations.

### Predictive value

When assessing clinically significant results alone – AMH results beneath the 10th percentile for the subject’s age and birth control status – the results from the shipped TAP device demonstrated 100% sensitivity and 100% specificity in replicating significant results from venipuncture. Because these failure rates are so low, precise assessment is impractical. The ADx card demonstrated only 57% sensitivity and 88% specificity prior to adjustment, improving to 100% sensitivity and 88% specificity after a best-case correction factor was applied. This difference in specificity was significant (95% CI 0.01, 0.23). While the TAP and adjusted ADx card showed equally robust performance in identifying true positives, these best-case numbers translate to only a 48% positive predictive value for a “<10th percentile” finding on the ADx card.

Practically, out of every 100 patients who undergo testing, the ADx card would be expected to produce roughly eleven (10.9) false positives – representing more than half (52%) of low results obtained. The proportion of ADx false positives in-sample (4 of 11, or 36%) was lower due to a higher than expected number of subjects with low AMH confirmed via venipuncture (19.5% in-sample vs. 10% expected). In instances of these false positives with ADx cards, readings varied from venipuncture results by 81, 59, 34, and 52%, respectively (in order of processing date). In each of these instances, AMH obtained via venipuncture placed a woman in the 25-75th percentile for her age group, but the result from the ADx card was incorrectly classified as <10th percentile. Total protein correction factors are unlikely to account for these clinically and statistically significant levels of variation. The TAP device, shipped or otherwise, obtained no false positives.

While the TAP (both shipped and non-shipped) demonstrated a slightly higher rate of no result (QNS) at 3.8% of tests performed vs. ADx’s 2.5%, the difference was small and not statistically significant (*p* = 0.81).

### Net promoter score (NPS) and pain scores

The study evaluated participant-reported pain using the NRS-11 and Net Promoter Score (NPS) in a post-draw survey that participants completed independently. Forty of the forty-one participants completed the survey after their draws.

**Fig. 4:**
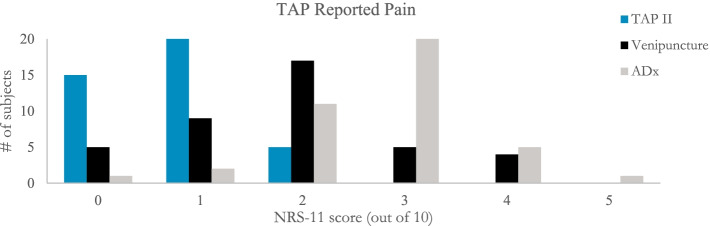
Surveyed pain distribution. Subject reported pain levels while using the TAP II Device on the NRS-11, a 0–10 pain scale with associated benchmarks to help respondents identify their relative levels of pain

The TAP averaged a 0.75 pain score while the ADx averaged 2.73 and venipuncture averaged 1.85. 100% of responding participants described the pain associated with the TAP as less than “very noticeable” (below 3 on the NRS-11) – with most (88%) rating it as painless or barely noticeable (a 0 or 1). In contrast, most (65%) of participants described the ADx as a 3 or greater, with an overall range of 0–5. No participant preferred the ADx over the TAP from a pain standpoint (Fig. 4).

The calculated NPS for the TAP experience was + 72. In comparison, the ADx card scored − 48 and venipuncture scored + 20. The difference in NPS between the TAP and ADx is a staggering 120 points (95% CI 90, 149); for perspective, the difference between a well-known 5-star hotel, (NPS of 75) and a roadside lodge chain (NPS of 4) is 71 points (18). 34 subjects preferred the TAP to the ADx from an NPS perspective. Only 1 subject preferred the ADx to the TAP.

### Harms

Of the 40 subjects who filled out the end of study survey, one subject reported concerns with both the TAP device and ADx card. That subject reported the finger prick used in the ADx card test had a pain level of 3–4 on a pain scale of 0–10 and that the pain lasted for 24 hours. The same subject also noted visible “rings” on her skin where the TAP devices had been placed, though reported the associated pain as only a ‘1’ out of 10. Neither report met criteria as a device adverse event.

Additionally, insufficient sampling for the small number of samples that precluded lab processing due to QNS demonstrates this possible risk of any at-home blood test. The real-world response to this would be to immediately offer repeat collection and testing to deliver interpretable results to the patient.

## Discussion

TAP results indicate clinical superiority and strong consumer preference over the on-market ADx card. The TAP did not generate any false positives that might have led to an unnecessary referral to an REI. The ADx card would have generated 4 such false-positive referrals to the clinic within the study population alone – implying a population positive predictive value of only 48% (versus 100% for the TAP). The TAP had an NPS of + 72, consistently low-to-no pain scores (< 3, range 0–2). The ADx card had an NPS of − 48 and a pain score four times that of the TAP.

This is the first study to directly compare multiple at-home collection products against venipuncture. This study demonstrates the strong concordance of AMH levels found in blood via standard venipuncture and self-administered at-home blood collection using the TAP device. Further, it shows that the TAP device is a significant improvement, in both accuracy and patient experience, from the on-market ADx cards, and can maintain high agreement with venipuncture even after shipment via commercial parcel services. The TAP device is more expensive (~$20 per-unit) than the ADx card (~$4.50 per-unit), however, this cost is offset by reduced laboratory processing complexity and cost compared to the ADx card. Additionally, the TAP device’s heightened accuracy and higher concordance with venipuncture may preclude the need for repeat laboratory testing. An economic analysis of these tradeoffs are beyond the scope of this paper.

The magnitude of the performance difference between devices was unexpected, as the ability of the ADx card to reproduce AMH results obtained via venipuncture with high reliability has been previously published. Several differences in methods may explain this discordance, including the duration between collection and testing, the exact total protein correction factor, and the assay used by the lab. Differences in the study populations and distributions of low AMH levels may also play a role. Of note, the discordance is apparent despite the ‘favorable’ treatment of the ADx card, as described above.

Strengths of the study include that it replicated real world shipping conditions and time to analysis which had not been reviewed in prior published literature. There was also comparison across the TAP device arm of both shipped and non-shipped samples for better comparison data. The simultaneous collection of venipuncture at the time of TAP and ADx collection and the cross enrollment of women across all arms allowed for a more direct comparison of results.

Limitations of this study include the diversity of the sample; even though the study met its recruitment goals for minority participation, the percent of subjects identifying as Black/African American and Hispanic were below that of the trial’s catchment area (Boston) [23]. Additionally, the age range for participants was constrained to patients less than 40 years old, potentially limiting generalizability in older patients. The sample size (*n* = 41) was insufficient to estimate precise sensitivity and specificity for the TAP device due to very low failure rates, but was sufficient to show dramatic superiority to the ADx card. Additionally, the “low AMH” values were underrepresented in the sample. Additional studies may be needed to ensure accuracy/validity at lower concentrations, given there were no samples with an AMH lower than 0.61 ng/dL. Assays other than the Roche Elecsys® used in this study may produce different results. Those results would have to be systematically different by blood collection method to invalidate these findings, which is possible but not predicted. This was not specifically examined in this study, and may benefit from future exploration. The correction factors used for both devices were based on within-sample data, and likely overfit and specific to the 72 hour processing time [16] – however the effect on the TAP device analysis was small and the effect on the ADx card was intentional as part of its favorable treatment. As noted elsewhere, the thresholds used for the definition of false positives were based on studies using different AMH assays and may vary in clinical practice, though results were not sensitive to thresholds chosen. Finally, as stated earlier, this study was funded by the sponsor, Turtle Health. However, to mitigate any potential bias that a sponsored study creates, all authors that were involved in study design and data analysis were compensated at fair market value for time contributed, and none have equity in the company. None of the authors are employees or board members of the company.

## Conclusions

Easily accessible and minimally invasive sampling through the TAP device allows for testing of Anti-Müllerian hormone levels that is sensitive, specific, and in concordance with results via venipuncture. The TAP device was not only more accurate than the alternative at-home serum AMH test via the ADx card, but also had significantly lower pain and higher patient satisfaction scores.

This modality will provide the growing number of patients receiving remote fertility counseling with increased access to accurate assessment of ovarian reserve. Further evaluation of the TAP II device for serum AMH testing could benefit from a larger sample size, more inclusive participant demographics, and use of alternative assays. Future studies could explore the TAP device’s accuracy in evaluating other hormones of interest, with the potential to significantly broaden the scope of this method for at-home testing.

## Supplementary Information

**Additional file 1: Figure 5.** Study Procedure. Head-to-head comparison of study procedure, including timeline and analysis between samples collected via venipuncture, shipped TAP, non-shipped TAP (stored) and ADx Card (stored) at t = 0 (within 6 hours of sample collection) and t = 72 hours. Note: TAP = TAP II Device.

## Data Availability

The datasets generated and/or analyzed during the current study are not publicly available out of extra precautions taken for patient information privacy, but are available in their de-identified from the corresponding author on reasonable request.

## Abbreviations

AMH: Anti-Mullerian hormone
TAP: TAP II device
REI: Reproductive endocrinology and infertility
AMH^2^: Anti-Müllerian Hormone – At My Home
SELF-HELP: Sonograms Enable Looking Forward - Home Examinations Led by Providers
CLIA: Clinical Laboratory Improvement Amendments
UPS: United Parcel Services
QNS: Quantity not sufficient
NPS: Net promoter score
IUD: Intrauterine device
RMSD: Root-mean-square deviation
