# Supplementary material for: Evaluation of at-home serum anti-Müllerian hormone testing: a head-to-head comparison study
Source: Reprod Biol Endocrinol. 2022 Sep 1;20:131. doi: 10.1186/s12958-022-01004-2 (PMC9434544; doi:10.1186/s12958-022-01004-2)
Supplement: Supplementary file 1 — Additional file 1: Figure 5. Study Procedure. Head-to-head comparison of study procedure, including timeline and analysis between samples collected via venipuncture, shipped TAP, non-shipped TAP (stored) and ADx Card (stored) at t = 0 (within 6 hours of sample collection) and t = 72 hours. Note: TAP = TAP II Device. [file 12958_2022_1004_MOESM1_ESM.docx]

**Supplemental Data**


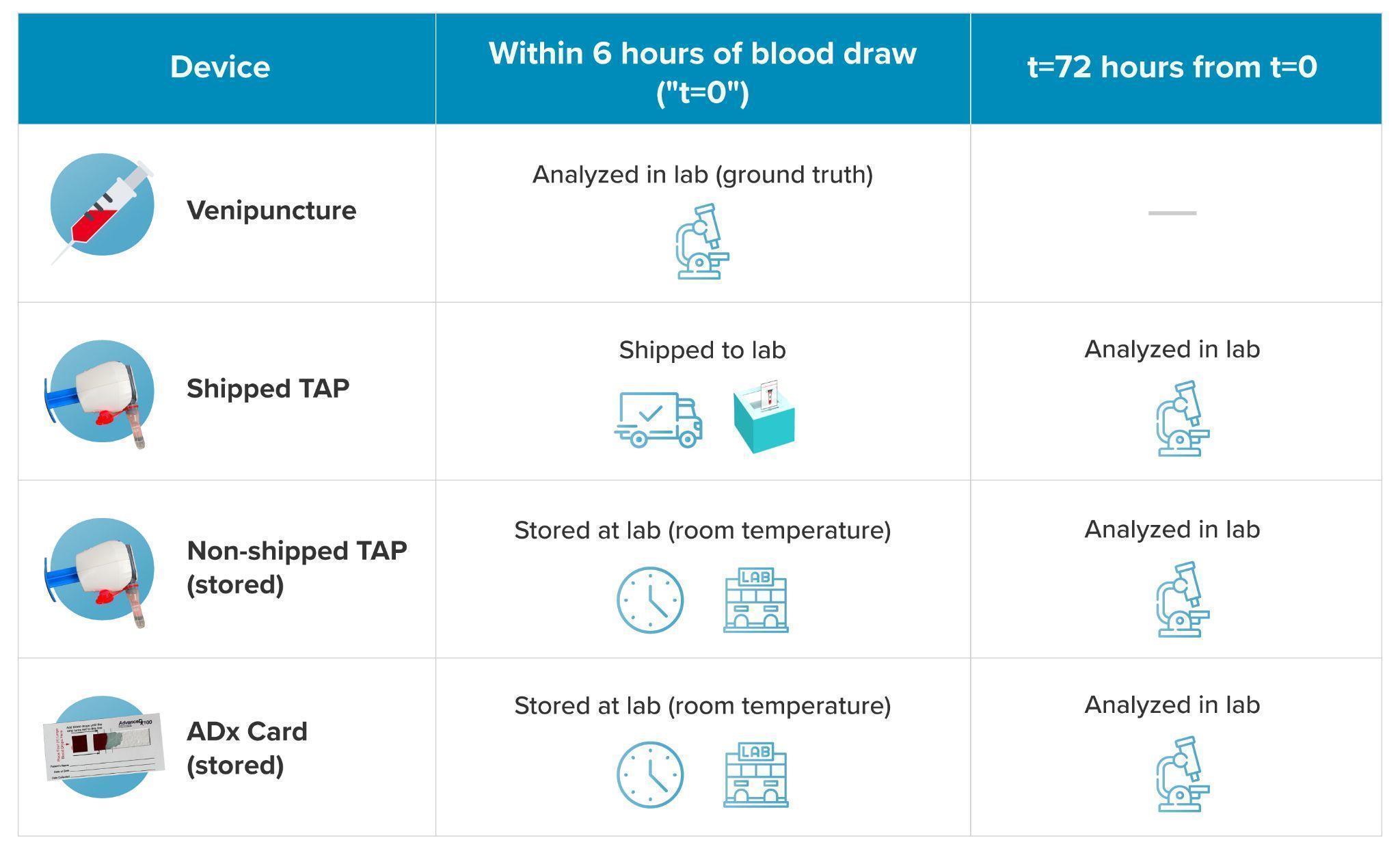


**Figure 5:** Study Procedure. Head-to-head comparison of study procedure, including timeline and analysis between samples collected via venipuncture, shipped TAP, non-shipped TAP (stored) and ADx Card (stored) at t=0 (within 6 hours of sample collection) and t=72 hours. Note: TAP = TAP II Device
